# Finite Element Simulation of Ionic Electrodiffusion in Cellular Geometries

**DOI:** 10.3389/fninf.2020.00011

**Published:** 2020-03-25

**Authors:** Ada J. Ellingsrud, Andreas Solbrå, Gaute T. Einevoll, Geir Halnes, Marie E. Rognes

**Affiliations:** ^1^Department for Scientific Computing and Numerical Analysis, Simula Research Laboratory, Oslo, Norway; ^2^Centre for Integrative Neuroplasticity, University of Oslo, Oslo, Norway; ^3^Department of Physics, University of Oslo, Oslo, Norway; ^4^Faculty of Science and Technology, Norwegian University of Life Sciences, Ås, Norway

**Keywords:** finite element, electrodiffusion, ion concentrations, cell membrane, ephaptic coupling, KNP-EMI

## Abstract

Mathematical models for excitable cells are commonly based on cable theory, which considers a homogenized domain and spatially constant ionic concentrations. Although such models provide valuable insight, the effect of altered ion concentrations or detailed cell morphology on the electrical potentials cannot be captured. In this paper, we discuss an alternative approach to detailed modeling of electrodiffusion in neural tissue. The mathematical model describes the distribution and evolution of ion concentrations in a geometrically-explicit representation of the intra- and extracellular domains. As a combination of the electroneutral Kirchhoff-Nernst-Planck (KNP) model and the Extracellular-Membrane-Intracellular (EMI) framework, we refer to this model as the KNP-EMI model. Here, we introduce and numerically evaluate a new, finite element-based numerical scheme for the KNP-EMI model, capable of efficiently and flexibly handling geometries of arbitrary dimension and arbitrary polynomial degree. Moreover, we compare the electrical potentials predicted by the KNP-EMI and EMI models. Finally, we study ephaptic coupling induced in an unmyelinated axon bundle and demonstrate how the KNP-EMI framework can give new insights in this setting.

## 1. Introduction

The most common computational models for excitable cells are those based on cable theory (Rall, 1977; Koch, 1999). In its standard form, the cable model is based on several simplifying assumptions, most importantly that the extracellular potential and both intracellular and extracellular ion concentrations are constant in space and time. Multi-compartmental neuron models based on cable theory are widely used within the field of neuroscience to simulate large network of interacting neurons (see e.g., Markram et al., 2015). In such models, only synaptic interactions between neurons are considered, whereas changes in the extracellular field and extracellular ion concentrations associated with a neuron's activity are assumed to be too small to have any influence on its neighboring neurons (or itself). Although these assumptions are only approximations, the resulting models still give accurate predictions of neuronal electrodynamics in many scenarios. Indeed, concentration changes are often limited by neuronal and glial uptake mechanisms that strive toward maintaining concentrations close to basal levels.

However, there are also many scenarios that involve dramatic changes in extracellular ion concentrations. On a large spatial scale, ion concentration changes are a trademark of several pathological conditions, such as spreading depression or epilepsy (Dietzel et al., 1989; Somjen, 2001; Syková and Nicholson, 2008; Ayata and Lauritzen, 2015). Extracellular concentration shifts will lead to changes in neuronal reversal potentials, and can thus affect the dynamical properties of the neurons (Kager et al., 2000; Øyehaug et al., 2012; Wei et al., 2014). Under non-pathological conditions, concentration-dependent, electrodiffusive effects are hypothesized to be important in specific microdomains of the brain (Savtchenko et al., 2017). In general, the extracellular ion concentration changes resulting from a neuronal event can be expected to be largest in regions where the extracellular space is small and confined.

Similarly, there are several scenarios where the assumption of a constant extracellular potential may be questionable. For instance, ephaptic interactions have been reported to play a role for neural phenomena taking place at both small and large spatial scales (Holt and Koch, 1999; Bokil et al., 2001; Anastassiou et al., 2011; Anastassiou and Koch, 2015; Goldwyn and Rinzel, 2016; Tveito et al., 2017b; Han et al., 2018; Shifman and Lewis, 2019). Ephaptic interaction (or coupling) is a coupling between neurons via the extracellular potential, which is hard or impossible to represent under the aforementioned assumption.

The olfactory nerve is one example in which variations in ion concentrations and extracellular potentials may be important. Whereas most axons in the mammalian brain are coated in an insulating layer of *myelin*, the axons in the olfactory nerve are unmyelinated and organized in tight bundles (Doucette, 1984; Griff et al., 2000). In view of the tight packing, one might expect large ion concentration variations in the extracellular space between the olfactory nerve axons. Moreover, the olfactory nerve axon arrangement will maximize any ephaptic coupling, with a potential evolutionary purpose (Lowe, 2003). In addition, diffusion along extracellular ion concentration gradients can generate so-called *diffusion potentials* (Halnes et al., 2016; Savtchenko et al., 2017; Solbrå et al., 2018), which may constitute an additional ephaptic effect on membrane potentials.

There are several computational studies considering ephaptic interaction in the brain. Bokil et al. (2001) use a simplified model based on cable theory, and find that an action potential in a single axon can evoke action potentials in neighboring axons. A more detailed model for coupling intra- and extracellular currents is the *Extracellular-Membrane-Intracellular* (EMI) model (Krassowska and Neu, 1994; Ying and Henriquez, 2007; Agudelo-Toro, 2012; Agudelo-Toro and Neef, 2013; Tveito et al., 2017a,b). The EMI model incorporates explicit 3D shapes of the neuron, allowing for morphologically detailed descriptions of the neuropil. However, neither of the aforementioned frameworks explicitly model the ion concentrations and can therefore not capture ephaptic effects due to electrodiffusion, such as diffusive potentials.

The most physically detailed scheme for modeling electrodiffusion is the *Poisson-Nernst-Planck* (PNP) framework (Lopreore et al., 2008; Pods et al., 2013; Holcman and Yuste, 2015; Cartailler et al., 2017a,b; Sacco et al., 2017). The PNP framework is based on explicitly simulating charge relaxation processes taking place at small spatiotemporal scales (~nm and ~ns), and thus requires high resolutions in both time and space. Consequently, applications have been limited to studying dynamics at the ion channel and cell membrane level. An alternative approach is to assume that the bulk tissue is electroneutral, thus circumventing the need for explicit modeling of charge relaxation processes. Models based on the electroneutrality assumption are therefore numerically stable for coarser spatial and temporal resolutions, allowing for longer simulations on larger domains.

On this background, a series of electroneutral models for ionic electrodiffusion have been developed, both for homogenized domains (Mori et al., 2008; Halnes et al., 2013, 2016, 2017; Niederer, 2013; Pods, 2017; Solbrå et al., 2018), and for domains including an explicit geometrical representation of the cells and of the extracellular space (Mori and Peskin, 2009). In particular, Mori and Peskin (2009) presents a finite volume method for solving a system of equations describing cellular electrical activity accounting for both geometrical effects and ion concentration dynamics.

In this paper, we present a variation of the Mori and Peskin (2009) model and introduce a mortar-based finite element formulation of this model. Key advantages of the finite element formulation are (i) the independence of dimension: the same scheme is applicable for one-, two-, or three-dimensional domains (with zero-, one-, or two-dimensional cell membranes/interfaces); (ii) the handling of complicated interface geometries; and (iii) the straightforward use of more accurate, i.e., higher order polynomial schemes. The framework can be viewed as a combination of the EMI framework and the electroneutral *Kirchhoff-Nernst-Planck* (KNP) framework (Solbrå et al., 2018), and will henceforth be referred to as the KNP-EMI framework. Previous numerical schemes for the KNP framework are restricted to simplified 1D geometries (Halnes et al., 2013, 2015; Sætra et al., 2020), or components within a hybrid modeling scheme to compute extracellular dynamics (Halnes et al., 2016, 2017; Solbrå et al., 2018).

The KNP-EMI framework can be viewed as an extension of the EMI framework by the explicit modeling of ion concentrations and the effects of ionic electrodiffusion. We here evaluate the effect of these extensions by comparing the KNP-EMI and EMI solutions in idealized axon domains, and find that the solutions are qualitatively similar but differ locally. However, the KNP-EMI simulations give further insights into the importance of extracellular bulk conductivities for ephaptic couplings in neural tissue: KNP-EMI simulations of idealized, unmyelinated axon bundles reveal increased extracellular bulk conductivities and, as a result, a reduced tendency toward induction of action potentials in neighboring axons.

## 2. Methods

We present the governing equations for ionic electrodiffusion in neural tissue with a geometrically explicit representation of the cellular membranes in section 2.1 below. To take full advantage of this framework, a numerical solution scheme capable of efficiently handling three-dimensional, complicated geometries is required. We here propose a novel numerical solution scheme using a mortar finite element method (Bernardi et al., 1993; Agudelo-Toro and Neef, 2013) and a two-step splitting scheme, described in section 2.2. This solution algorithm flexibly allows for arbitrary geometries and efficient solution of the separate subproblems. Our implementation of this algorithm is openly available at: https://zenodo.org/record/3492075#.XahQOhh9g5k.

### 2.1. A Mathematical Framework for Electrodiffusion With Explicit Membrane Representation

#### 2.1.1. Representation of the Computational Domain

**Figure 1:**
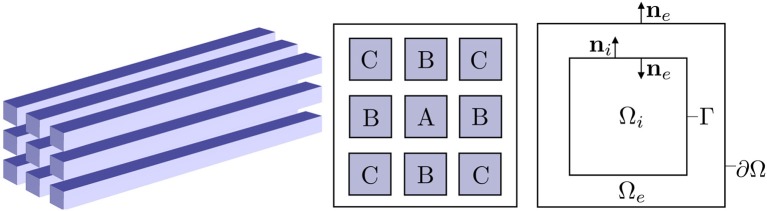
Overview of the computational domains. **(Left)** Idealized axon bundle consisting of 9 cuboid-shaped axons. **(Middle)** Cross-section of the axon bundle, where the axons are labeled with repeated labels for symmetric positions. **(Right)** Idealized 2D computational domain with one intracellular region Ω_*i*_ and extracellular region Ω_*e*_.

We consider *N* domains $\Omega_{i^{n}}\subset {\mathbb{R}}^{d}$ (*d* = 1, 2, 3) for *n* = 1, …, *N* representing disjoint intracellular regions (physiological cells, e.g., neurons) and an extracellular region Ω_*e*_, and let the complete domain $\Omega =\Omega_{i^{1}}\cup \cdots \cup \Omega_{i^{N}}\cup \Omega_{e}$ with boundary ∂Ω. See Figure 1 (Right) for an illustration of a sample domain configuration. We denote the cell membrane associated with cell *i*^*n*^, i.e., the boundary of the physiological cell $\Omega_{i^{n}}$, by Γ_*n*_. We assume that Γ_*n*_ ∩ Γ_*m*_ = ∅ for all *n*≠*m* and that Γ_*n*_ ∩ ∂Ω = ∅ (It follows that $\partial \Omega_{i^{n}}\cap \partial \Omega_{e}=\emptyset$ for all *n* = 1, …, *N*.). For simplicity and clarity, we present the mathematical model for one intracellular region $\Omega_{i^{1}}=\Omega_{i}$ with membrane Γ below. The extension to multiple intracellular regions is immediate (but notationally cumbersome).

#### 2.1.2. Intracellular and Extracellular Governing Equations

We will here derive a system of coupled, time-dependent, non-linear partial differential equations to describe ionic electrodiffusion in this domain. We consider a set of ion species *K*. Typically *K* will include sodium *Na*^+^, potassium *K*^+^, and chloride *Cl*^-^. For each ion species *k* ∈ *K* and each region *r* ∈ {*i, e*}, we model the *ion concentrations* [*k*]_*r*_ : Ω_*r*_ × (0, *T*] → ℝ (mol/m^3^) and the *electrical potentials* ϕ_*r*_ : Ω_*r*_ × (0, *T*] → ℝ (V). Conservation of ions for the bulk of each region Ω_*r*_ stipulates that

(1) $$\begin{array}{l} \frac{\partial {\left[k\right]}_{r}}{\partial t}+\nabla \cdot {\text{J}}_{r}^{k}=0\text{ in}\Omega_{r},\text{ for }r\in \left\{i,e\right\}, \end{array}$$

for *t* ∈ (0, *T*]. Here, ${\text{J}}_{r}^{k}:\Omega_{r}\times \left(0,T\right]\to {\mathbb{R}}^{d}$ is the regional ion flux density [mol/(m^2^s)] of ion *k*. To proceed, we invoke the KNP assumption of bulk electroneutrality. In this case, the ion flux densities ${\text{J}}_{r}^{k}$ satisfy:

(2) $$\begin{array}{l} -F\underset{k\in K}{\sum }z^{k}\nabla \cdot {\text{J}}_{r}^{k}=0\text{ in}\Omega_{r},\text{ for }r\in \left\{i,e\right\}, \end{array}$$

where *z*^*k*^ is the valence of ion species *k* and *F* is Faraday's constant. The assumption (2) states that the total net flow of ions (weighted by the respective valences) out of any infinitesimal representative bulk volume is zero. Furthermore, we assume that the each regional ion flux density can be expressed by a Nernst-Planck equation as follows:

(3) $$\begin{array}{l} {\text{J}}_{r}^{k}=-D_{r}^{k}\nabla {\left[k\right]}_{r}-\frac{D_{r}^{k}z^{k}}{\psi }{\left[k\right]}_{r}\nabla \phi_{r},\text{ in}\Omega_{r},\;r\in \left\{i,e\right\}. \end{array}$$

Here, $D_{r}^{k}$ denotes the effective diffusion coefficient (m^2^/s) of ion species *k* in the region *r*. The constant ψ = *RTF*^−1^ combines Faraday's constant *F*, the absolute temperature *T*, and the gas constant *R*. The ion flux density, i.e., the flow rate of ions per unit area, is thus modeled as the sum of two terms: (i) the diffusive movement of ions due to ionic gradients $-D_{r}^{k}\nabla {\left[k\right]}_{r}$ and (ii) the ion concentrations that are transported via electrical potential gradients, i.e., the ion migration $-D_{r}^{k}z^{k}\psi^{-1}{\left[k\right]}_{r}\nabla \phi_{r}$ where $D_{r}^{k}\psi^{-1}$ is the electrochemical mobility. This model ignores convective effects, and thus assumes that the underlying material (typically fluid) is at rest. As the potential ϕ_*e*_ is only determined up to a constant in Equations (1–3)„ an additional constraint is required, e.g.,

(4) $$\begin{array}{l} \int_{\Omega_{e}}\phi_{e}\text{d}x=0. \end{array}$$

By inserting (3) into (2) we recognize (from volume conductor theory) the following expression for the bulk conductivity σ_*r*_:

(5) $$\begin{array}{l} \sigma_{r}=\frac{F}{\psi }\underset{k\in K}{\sum }D_{r}^{k}{\left[k\right]}_{r}{\left(z^{k}\right)}^{2}. \end{array}$$

Notably, the bulk conductivity σ_*r*_ depends on the ion concentrations [*k*]_*r*_ and the diffusion coefficients $D_{r}^{k}$. Electrodiffusive models without explicit modeling of the ion concentrations typically set the bulk conductivity as a independent parameter (e.g., Krassowska and Neu, 1994; Bokil et al., 2001; Tveito et al., 2017a).

Inserting (3) into (1) and into (2), we thus obtain a system of *N*|*K*|+*N* equations (*N*|*K*| parabolic, *N* elliptic) for the *N*|*K*|+*N* unknown scalar fields. The system remains to be closed by appropriate initial conditions, boundary conditions, and importantly interface conditions.

#### 2.1.3. Interface Conditions

We next turn to modeling the cell membrane currents and membrane potential across the interface Γ. We denote the membrane potential ϕ_*M*_ as the jump in the electrical potential over the membrane:

(6) $$\begin{array}{l} \phi_{M}=\phi_{i}-\phi_{e}\text{ on }\Gamma . \end{array}$$

We introduce the *total ionic current density* *I*_*M*_ : Γ × (0, *T*) → ℝ [C/(m^2^s)] across the interface Γ. By definition and by conservation of total charge, we have that

(7) $$\begin{array}{l} I_{M}\equiv -F\underset{k\in K}{\sum }z^{k}{\text{J}}_{e}^{k}\cdot {\text{n}}_{e}=F\underset{k\in K}{\sum }z^{k}{\text{J}}_{i}^{k}\cdot {\text{n}}_{i}. \end{array}$$

where **n**_*r*_ denotes the boundary normal pointing out of Ω_*r*_ for *r* ∈ {*i, e*}. Next, we assume that *I*_*M*_ consists of two components: (i) a total channel current *I*_ch_ and (ii) a capacitive current *I*cap:

(8) $$\begin{array}{l} I_{M}=I_{\text{ch}}+I_{\text{cap}}. \end{array}$$

We further assume that the total channel current *I*_ch_ is the sum of the ion specific channel currents ${\text{I}}_{\text{ch}}^{k}$:

(9) $$\begin{array}{l} I_{\text{ch}}=\underset{k\in K}{\sum }I_{\text{ch}}^{k},\;I_{\text{ch}}^{k}=I_{\text{ch}}^{k}\left(\phi_{M},{\left[k\right]}_{\cdot },\ldots \right). \end{array}$$

The channel currents ${\text{I}}_{\text{ch}}^{k}$ are subject to modeling. Typical models for ${\text{I}}_{\text{ch}}^{k}$ notably includes an synaptic input current *I*syn, leaky passive neuron, Hodgkin-Huxley etc., and will be detailed further below in section 2.1.4. On the other hand, the capacitive current *I*cap is defined over to be the capacitance *C*_*M*_ times the rate of change of the voltage (Sterratt et al., 2011), hence:

(10) $$\begin{array}{l} I_{\text{cap}}=C_{M}\frac{\partial \phi_{M}}{\partial t}. \end{array}$$

Inserting (10) into (8) and rearranging gives the following relation for the membrane potential ϕ_*M*_:

(11) $$\begin{array}{l} \frac{\partial \phi_{M}}{\partial t}=\frac{1}{C_{M}}\left(I_{M}-I_{\text{ch}}\right). \end{array}$$

It remains to specify a set of interface conditions for the specific ion fluxes ${\text{J}}_{r}^{k}\cdot {\text{n}}_{r}$ for *r* ∈ {*i, e*}. Here, we propose a heuristic approach via ion specific capacitive current modeling. An alternative approach is presented in Mori and Peskin (2009). As for the total current, we assume that the capacitive current can be represented as a sum of ion-associated currents:

(12) $$\begin{array}{l} I_{\text{cap}}=\underset{k\in K}{\sum }I_{\text{cap}}^{k}. \end{array}$$

Without loss of generality, we let the ion specific capacitive current *I*cap, *r*^*k*^ in region Ω_*r*_ at the interface Γ be some fraction $\alpha_{r}^{k}$ of the total capacitive current *I*cap:

(13) $$\begin{array}{l} I_{\text{cap},r}^{k}=\alpha_{r}^{k}I_{\text{cap}}. \end{array}$$

Specifically, we assume that:

(14) $$\begin{array}{l} \alpha_{r}^{k}=\frac{D_{r}^{k}{\left(z^{k}\right)}^{2}{\left[k\right]}_{r}}{\sum_{l\in K}D_{r}^{l}{\left(z^{l}\right)}^{2}{\left[l\right]}_{r}}, \end{array}$$

and note that $\underset{k\in K}{\sum }\alpha_{r}^{k}=1$ for *r* ∈ {*i, e*}. By definition of the ion currents and the expression for the capacitive current given by (8), we let the intracellular and extracellular ion fluxes across the membrane be given by:

(15) $$\begin{array}{l} {\text{J}}_{i}^{k}\cdot {\text{n}}_{i}=\frac{I_{\text{ch}}^{k}+\alpha_{i}^{k}\left(I_{M}-I_{\text{ch}}\right)}{Fz^{k}},\;-{\text{J}}_{e}^{k}\cdot {\text{n}}_{e}=\frac{I_{\text{ch}}^{k}+\alpha_{e}^{k}\left(I_{M}-I_{\text{ch}}\right)}{Fz^{k}}, \end{array}$$

for *k* ∈ *K*.

#### 2.1.4. Modeling Specific Ion Channels

The framework presented thus far allows for general representations of the ion channel current dynamics. In particular, the framework admits different choices of ion specific channel current models ${\text{I}}_{\text{ch}}^{k}$. An advantage of the geometrically explicit framework is that it allows for different channel currents models for individual cells and e.g., geometrically heterogeneous material properties. We here summarize two examples of ion specific channel currents: a passive membrane model (Sterratt et al., 2011) and the Hodgkin-Huxley model (Hodgkin and Huxley, 1952).

##### 2.1.4.1. Passive membrane dynamics

We model the passive membrane channel current for ion species *k* as (Sterratt et al., 2011):

(16) $$\begin{array}{l} I_{\text{ch}}^{k}\left(\phi_{M}\right)=g_{L}^{k}\left(\phi_{M}-E^{k}\right), \end{array}$$

where $g_{L}^{k}$ is a constant leak conductivity, and *E*^*k*^ is the ion specific reversal potential, given by

$$\begin{array}{l} E^{k}=\frac{RT}{z^{k}F}\ln\frac{{\left[k\right]}_{e}}{{\left[k\right]}_{i}}, \end{array}$$

with valence *z*^*k*^, Faraday's constant *F*, absolute temperature *T*, and gas constant *R*.

##### 2.1.4.2. Hodgkin-Huxley membrane dynamics

In order to model active membrane dynamics, we use the standard Hodgkin-Huxley membrane model (Hodgkin and Huxley, 1952). The ion species under consideration are sodium Na^+^, potassium K^+^, and chloride Cl^-^, and the model additionally introduces three gating variables *m, h, n* associated with sodium channel activation, potassium channel activation, and potassium channel inactivation, respectively. The membrane potential ϕ_*M*_ is then modeled by the following specialization of (11):

(17) $$\begin{array}{l} \frac{\partial \phi_{M}}{\partial t}=\frac{1}{C_{M}}\left(I_{M}-I_{\text{ch}}^{\text{Na}}-I_{\text{ch}}^{\text{K}}-I_{\text{ch}}^{\text{Cl}}\right), \end{array}$$

with ion specific membrane channel currents:

(18) $$\begin{array}{l} I_{\text{ch}}^{\text{Na}}\left(\phi_{M}\right)={ḡ}^{\text{Na}}m^{3}h\left(\phi_{M}-E^{\text{Na}}\right), \end{array}$$

(19) $$\begin{array}{l} I_{\text{ch}}^{\text{K}}\left(\phi_{M}\right)={ḡ}^{\text{K}}n^{4}\left(\phi_{M}-E^{\text{K}}\right), \end{array}$$

(20) $$\begin{array}{l} I_{\text{ch}}^{\text{Cl}}\left(\phi_{M}\right)={ḡ}^{\text{Cl}}\left(\phi_{M}-E^{\text{Cl}}\right). \end{array}$$

Here, ḡ^*k*^ is the maximal conductivity for ion species *k*. The gating variables are governed by the following ODE:

(21) $$\begin{array}{l} \frac{\partial p}{\partial t}=\alpha_{p}\left(\phi_{M}\right)\left(1-p\right)-\beta_{p}\left(\phi_{M}\right)p, \end{array}$$

for *p* ∈ {*m, h, n*}. The rate constants α_*p*_ and β_*p*_ take the form

(22) $$\begin{array}{l} \alpha_{p}\left(\phi_{M}\right)=p_{\infty }\left(\phi_{M}\right)/\tau_{p}, \end{array}$$

(23) $$\begin{array}{l} \beta_{p}\left(\phi_{M}\right)=\left(1-p_{\infty }\left(\phi_{M}\right)\right)/\tau_{p}, \end{array}$$

where *p*_∞_ is the steady state value for activation and τ_*p*_ is the time constant.

#### 2.1.5. Initial and Boundary Conditions

We assume that initial conditions are given for all ion concentrations, both intracellularly and extracellularly:

(24) $$\begin{array}{l} {\left[k\right]}_{r}\left(x,0\right)={\left[k\right]}_{r}^{0}\left(x\right)\;x\in \Omega_{r},\;r\in \left\{i,e\right\}. \end{array}$$

Furthermore, we assume that these conditions are compatible with the assumption of bulk electroneutrality, i.e., that the initial state of the system satisfies:

(25) $$\begin{array}{l} \underset{k\in K}{\sum }z^{k}{\left[k\right]}_{e}^{0}=0. \end{array}$$

In addition, we assume that an initial condition is given for the membrane potential:

(26) $$\begin{array}{l} \phi_{M}\left(x,0\right)=\phi_{M}^{0}\left(x\right),\;x\in \Gamma . \end{array}$$

Finally, a set of boundary conditions will close the system. We describe specific boundary conditions in the numerical experiments in section 2.3.

#### 2.1.6. Summary of Governing Equations

In summary, the mathematical framework for electrodiffusion with explicit geometrical representation of the cell membranes is comprised of the bulk equations (1), (2) with (3), the interface conditions (7), (11) with (6) and (9), and (15) with (14), the initial conditions (24) and (26), and additional boundary conditions. We will refer to this set of equations as the KNP-EMI framework.

### 2.2. Numerical Methods

To solve the KNP-EMI framework numerically, we consider a finite difference time integration scheme, a splitting scheme, and a mortar finite element method in space. We derive the new finite element scheme and describe the splitting algorithm in the sections below.

#### 2.2.1. Weak Formulation of the Governing Equations

Multiplying (1) with test functions $v_{r}^{k}$ (for *r* ∈ {*i, e*}), integration over the intracellular and extracellular domains Ω_*i*_ and Ω_*e*_ separately, integration by parts, and inserting (15) for the ion fluxes across the membrane, yields

(27) $$\begin{array}{l} \int_{\Omega_{i}}\frac{\partial {\left[k\right]}_{i}}{\partial t}v_{i}^{k}-{\text{J}}_{i}^{k}\cdot \nabla v_{i}^{k}\text{ d}x+\frac{1}{Fz^{k}}\int_{\Gamma }\left(I_{\text{ch}}^{k}+\alpha_{i}^{k}\left(I_{M}-I_{\text{ch}}\right)\right)v_{i}^{k}\text{ d}s=0, \end{array}$$

(28) $$\begin{array}{l} \int_{\Omega_{e}}\frac{\partial {\left[k\right]}_{e}}{\partial t}v_{e}^{k}-{\text{J}}_{e}^{k}\cdot \nabla v_{e}^{k}\text{ d}x-\frac{1}{Fz^{k}}\int_{\Gamma }\left(I_{\text{ch}}^{k}+\alpha_{e}^{k}\left(I_{M}-I_{\text{ch}}\right)\right)v_{e}^{k}\text{ d}s\\ =-\int_{\partial \Omega }{\text{J}}_{e}^{k}\cdot {\text{n}}_{e}v_{e}^{k}\text{ d}s. \end{array}$$

Similarly, multiplying (2) by test functions *w*_*r*_ for *r* ∈ {*i, e*}, integration by parts and inserting (7) for the total membrane current, yields

(29) $$\begin{array}{l} F\underset{k\in K}{\sum }z^{k}\int_{\Omega_{i}}{\text{J}}_{i}^{k}\cdot \nabla w_{i}\text{ d}x-\int_{\Gamma }I_{M}w_{i}\text{ d}s=0, \end{array}$$

(30) $$\begin{array}{l} F\underset{k\in K}{\sum }z^{k}\int_{\Omega_{e}}{\text{J}}_{e}^{k}\cdot \nabla w_{e}\text{ d}x+\int_{\Gamma }I_{M}w_{e}\text{ d}s=F\underset{k\in K}{\sum }z^{k}\int_{\partial \Omega }{\text{J}}_{e}^{k}\cdot {\text{n}}_{e}w_{e}\text{ d}s. \end{array}$$

The zero average constraint (4) is enforced by introducing an additional unknown (a Lagrange multiplier) *c*_*e*_ ∈ ℝ along with a test function *d*_*e*_ ∈ *R* and letting

(31) $$\begin{array}{l} \int_{\Omega_{e}}\phi_{e}d_{e}\text{ d}x=0. \end{array}$$

Finally, multiplying (11) by a test function *q*, and integrating over Γ yields

(32) $$\begin{array}{l} C_{M}\int_{\Gamma }\frac{\partial \left(\phi_{i}-\phi_{e}\right)}{\partial t}q\text{ d}s-\int_{\Gamma }\left(I_{M}-I_{\text{ch}}\right)q\text{ d}s=0. \end{array}$$

We remark that this is a weak formulation of a set of time-dependent, non-linear equations. In particular, recall that *I*_ch_ and ${\text{I}}_{\text{ch}}^{k}$ depend on ϕ_*M*_ and [*k*]_*r*_ cf. (9) while $\alpha_{r}^{k}$ depends on [*k*]_*r*_ cf. (14).

To solve this system numerically, we consider the following approximations.

- We discretize the time derivatives in (27)–(28) and (32) using a finite difference method.
- We approximate ${\text{J}}_{r}^{k}$ at time *t*^*n*^ by the linearized ion flux density [cf. (3)]:
  $$\begin{array}{l} {\text{J}}_{r}^{k}\approx -D_{r}^{k}\nabla {\left[k\right]}_{r}^{n}-\frac{D_{r}^{k}z^{k}}{\psi }{\left[k\right]}_{i}^{n-1}\nabla \phi_{i}^{n}. \end{array}$$
- We evaluate $\alpha_{r}^{k}$ at time *t*^*n*^ by the previous value [cf. (14)]:
  (33) $$\begin{array}{l} \alpha_{r}^{k}\approx \frac{D_{r}^{k}{\left(z^{k}\right)}^{2}{\left[k\right]}_{r}^{n-1}}{\sum_{l\in K}D_{r}^{l}{\left(z^{l}\right)}^{2}{\left[l\right]}_{r}^{n-1}}. \end{array}$$

Moreover, we evaluate *I*_ch_ and the discretization of (32) depending on the choice of ion channel model (cf. section 2.1.4) as follows.

- For the passive model, we insert the linear relation given by (16) directly in (27)–(28) and (32). Moreover, the implicit discretization of (32) reads as:
  (34) $$\begin{array}{l} \frac{\partial \left(\phi_{i}-\phi_{e}\right)}{\partial t}\approx \Delta t^{-1}\left(\phi_{M}^{n}-\phi_{M}^{n-1}\right), \end{array}$$
  at time *t*^*n*^ with $\phi_{M}^{n}=\phi_{i}^{n}-\phi_{e}^{n}$ and Δ*t* = *t*^*n*^ − *t*^*n*−1^.
- For the Hodgkin-Huxley model, we use the following two-step splitting procedure. Consider *n* ∈ [1, …, *N*] with *t*^*n*^ − *t*^*n*−1^ = Δ*t*, and assume that ${\left[k\right]}_{r}^{n-1}$ and $\phi_{M}^{n-1}$ at time step *t*^*n*−1^ are known.
  - In the first (ODE) step, we update the membrane potential $\phi_{M}^{n}$ at time step *t*^*n*^ by solving the ODE system (17)–(23), with *I*_*M*_ set to zero, using 25 explicit (forward) Euler steps of size Δt^*^ = Δ*t*/25.
  - In the second (PDE) step, we solve for ${\left[k\right]}_{r}^{n}$, $\phi_{r}^{n}$ and $I_{M}^{n}$ (for *r* ∈ {*i, e*}) in the linear system arising from spatial discretization of (27)–(32), with *I*_ch_ set to zero in (32), and *I*_ch_ approximated by
    (35) $$\begin{array}{l} I_{\text{ch}}\approx I_{\text{ch}}\left(\phi_{M}^{n},{\left[k\right]}_{\ldots }^{n-1}\right), \end{array}$$
    in (27)–(28), where $\phi_{M}^{n}$ is the membrane potential solution at *t*^*n*^ from the ODE step (see section 2.2.2 for details). The implicit discretization of (32) reads as:
    (36) $$\begin{array}{l} \frac{\partial \left(\phi_{i}-\phi_{e}\right)}{\partial t}\approx \Delta t^{-1}\left(\phi_{M}^{n}-\phi_{M}^{n-1}\right), \end{array}$$
    where $\phi_{M}^{n}=\phi_{i}^{n}-\phi_{e}^{n}$ is the membrane potential solution at *t*^*n*^ from the ODE step.

The steps are repeated until global end time *t*^*N*^ is reached.

#### 2.2.2. Spatial Discretization

**Figure 2:**
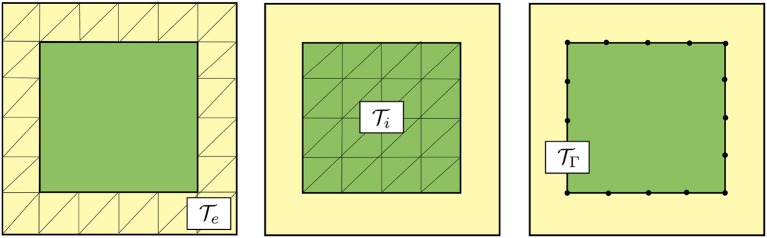
Schematic representation of meshes for the discretization of the PDE part of the governing electrodiffusive equations using a mortar finite element method. Mesh $T_{e}$ of the extracellular subdomain Ω_*e*_ **(left)**, mesh $T_{i}$ of the intracellular subdomain Ω_*i*_ **(middle)** and mesh $T_{\Gamma }$ of the interface Γ **(right)**. Note that the shared facets of the extracellular and intracellular meshes form the (codimension 1) mesh of the interface.

To numerically solve the PDE part of the governing equations defined on the domain Ω = Ω_*i*_∪Ω_*e*_, we use a mortar finite element method. We discretize each subdomain Ω_*r*_ by a conforming mesh $T_{r}$ for *r* ∈ {*i, e*}. We assume that the meshes $T_{i}$ and $T_{e}$ match at the common interface Γ, and define a (lower-dimensional) mesh $T_{\Gamma }$ of this interface (cf. Figure 2).

Next, we introduce separate finite element spaces for approximating the unknown fields in the weak formulation (27)–(32), [*k*]_*r*_ : Ω_*r*_ → ℝ, ϕ_*r*_ : Ω_*r*_ → ℝ for *r* ∈ {*i, e*}, *I*_*m*_ : Γ → ℝ. We approximate the ion concentrations [*k*]_*r*_ and potentials ϕ_*r*_ using continuous piecewise linear polynomials (linear Lagrange finite elements) over the meshes $T_{r}$. These fields thus have degrees of freedom defined on the vertices of the extracellular and intracellular meshes. The Lagrange multiplier *c*_*e*_ is approximated using a single real number. Furthermore, the transmembrane current *I*_*M*_ is approximated using continuous piecewise linear polynomials over the facet mesh $T_{\Gamma }$. We denote the finite element spaces for approximating [*k*]_*r*_ by $V_{r}^{k}$, the spaces for approximating ϕ_*r*_ by *W*_*r*_ and the spaces for approximating *I*_*M*_ by *Q*. Let ${\langle u,v\rangle }_{\Omega }=\int_{\Omega }uv\text{ d}x$. For notational simplicity, we denote the approximation of [*k*]_*r*_ by [*k*]_*r*_, the approximation of ϕ_*r*_ by ϕ_*r*_, and the approximation of *I*_*M*_ by *I*_*M*_ below. We here use linear polynomials for concreteness, but the formulation also applies directly for higher order polynomials.

We then solve the PDE step in the two-step splitting scheme described in section 2.2.1 as follows: given ${\left[k\right]}_{r}^{n}\in V_{r}^{k}$ and $\phi_{M}^{n}\in Q$ at time step *t*^*n*^, and the previously computed ${\text{I}}_{\text{ch}}^{k}$ and α^*k*^ [cf. (35) and (33)], find the ion concentrations ${\left[k\right]}_{r}\in V_{r}^{k}$, the potentials ϕ_*r*_ ∈ *W*_*r*_, the total transmembrane current density *I*_*M*_ ∈ *Q* at time step *t*^*n*+1^ (and the Lagrange multiplier *c*_*e*_ ∈ ℝ) such that:

$$\begin{array}{l} \frac{1}{\Delta t}{\langle {\left[k\right]}_{i},v_{i}^{k}\rangle }_{\Omega_{i}}-{\langle {\text{J}}_{i}^{k},\nabla v_{i}^{k}\rangle }_{\Omega_{i}}+{\langle \frac{\alpha_{i}^{k}}{Fz^{k}}I_{M},v_{i}^{k}\rangle }_{\Gamma }\\ =\frac{1}{\Delta t}{\langle {\left[k\right]}_{i}^{n},v_{i}^{k}\rangle }_{\Omega_{i}}-{\langle \frac{I_{\text{ch}}^{k}-\alpha_{i}^{k}I_{\text{ch}}}{Fz^{k}},v_{i}^{k}\rangle }_{\Gamma },\\ \frac{1}{\Delta t}{\langle {\left[k\right]}_{e},v_{e}^{k}\rangle }_{\Omega_{e}}-{\langle {\text{J}}_{e}^{k},\nabla v_{e}^{k}\rangle }_{\Omega_{e}}-{\langle \frac{\alpha_{e}^{k}}{Fz^{k}}I_{M},v_{e}^{k}\rangle }_{\Gamma }\\ =\frac{1}{\Delta t}{\langle {\left[k\right]}_{e}^{n},v_{e}^{k}\rangle }_{\Omega_{e}}+{\langle \frac{I_{\text{ch}}^{k}-\alpha_{e}^{k}I_{\text{ch}}}{Fz^{k}},v_{e}^{k}\rangle }_{\Gamma }\\ -{\langle {\text{J}}_{e}^{k}\cdot {\text{n}}_{e},v_{e}^{k}\rangle }_{\partial \Omega },F\underset{k\in K}{\sum }z^{k}{\langle {\text{J}}_{i}^{k},\nabla w_{i}\rangle }_{\Omega_{i}}\\ -{\langle I_{M},w_{i}\rangle }_{\Gamma }=0,\\ F\underset{k\in K}{\sum }z^{k}{\langle {\text{J}}_{e}^{k},\nabla w_{e}\rangle }_{\Omega_{e}}+{\langle c_{e},w_{e}\rangle }_{\Omega_{e}}+{\langle I_{M},w_{e}\rangle }_{\Gamma }\\ =F\underset{k\in K}{\sum }z^{k}{\langle {\text{J}}_{e}^{k}\cdot {\text{n}}_{e},w_{e}\rangle }_{\partial \Omega },\\ {\langle \phi_{e},d_{e}\rangle }_{\Omega_{e}}=0,\\ \frac{1}{\Delta t}{\langle \phi_{i}-\phi_{e},q\rangle }_{\Gamma }-\frac{1}{C_{M}}{\langle I_{M},q\rangle }_{\Gamma }=\frac{1}{\Delta t}{\langle \phi_{M}^{n},q\rangle }_{\Gamma }, \end{array}$$

for all $v_{i}^{k}\in V_{i}^{k}$, $v_{e}^{k}\in V_{e}^{k}$, *w*_*i*_ ∈ *W*_*i*_, *w*_*e*_ ∈ *W*_*e*_, *d*_*e*_ ∈ ℝ and *q* ∈ *Q*. The ion flux terms on the right-hand side are replaced by appropriate boundary conditions in the subsequent sections.

To evaluate the accuracy of the numerical solutions defined over Ω_*r*_ for *r* ∈ {*i, e*}, we use the standard *L*^2^ and *H*^1^ norms denoted by ||·||_0_ and ||·||_1_, respectively: for *u* : Ω_*r*_ → ℝ,

$$\begin{array}{l} ||u|{|}_{0}^{2}=\int_{\Omega_{r}}u^{2}\text{ d}x,\;||u|{|}_{1}^{2}=\int_{\Omega_{r}}u^{2}+\nabla u\cdot \nabla u\text{ d}x. \end{array}$$

In addition, for *I* : Γ → ℝ, we define the broken *L*^2^-norm by summing over the *L*^2^-norms over the mesh cells of the interface mesh $T_{\Gamma }$:

$$\begin{array}{l} ||I|{|}_{0,\Gamma }^{2}=\underset{f\in T_{\Gamma }}{\sum }||I{|}_{f}|{|}_{0}^{2}. \end{array}$$

#### 2.2.3. Implementation

The numerical scheme was implemented using a mixed dimensional framework from the FEniCS finite element library (Alnæs et al., 2015). The linear systems arising in the numerical experiments were solved using a direct (MUMPS) solver. The code is publicly available at: https://zenodo.org/record/3492075#.XahQOhh9g5k.

#### 2.2.4. Comparison With EMI Framework

In the numerical experiments comparing the KNP-EMI and the EMI models, the EMI model is discretized using the mortar finite element formulation as presented in Tveito et al. (2017b).

### 2.3. Computational Models and Parameters

We consider two model set-ups for testing the presented methodology (Model A and B), a model (Model C) for comparing simulation results between the KNP-EMI and EMI frameworks, and a model for studying ephaptic coupling (Model D). The model set-ups are described in detail here. The model parameters are given in Table 1, unless otherwise stated in the text. We assume that all axons in each simulation have the same membrane channel current *I*_ch_. We denote the spatial coordinates in this and subsequent sections by (*x, y, z*).

**Table 1:** The physical parameters and initial values used in the simulations.

**Parameter**	**Symbol**	**Value**	**Unit**	**References**
Gas constant	*R*	8.314	J/(K mol)	
Temperature	*T*	300	K	
Faraday's constant	*F*	9.648·10^4^	C/mol	
Membrane capacitance	*C*_*M*_	0.01	F/m	
Na^+^ diffusion coefficient	$D_{r}^{\text{Na}}$	1.33·10^−9^	m^2^/s	Hille, 2001
K^+^ diffusion coefficient	$D_{r}^{\text{K}}$	1.96·10^−9^	m^2^/s	Hille, 2001
Cl^-^ diffusion coefficient	$D_{r}^{\text{Cl}}$	2.03·10^−9^	m^2^/s	Hille, 2001
Na^+^ leak conductivity	$g_{L}^{\text{Na}}$	2.0	S/m^2^	
K^+^ leak conductivity	$g_{L}^{\text{K}}$	8.0	S/m^2^	
Cl^-^ leak conductivity	$g_{L}^{\text{Cl}}$	0	S/m^2^	
K^+^ HH max conductivity	ḡ^K^	360	S/m^2^	Hodgkin and Huxley, 1952
Na^+^ HH max conductivity	ḡ^Na^	1200	S/m^2^	Hodgkin and Huxley, 1952
Synaptic time constant	α	1.0·10^−3^	s	
Initial intracellular Na^+^ concentration	${\left[\text{Na}\right]}_{i}^{0}$	12	mM	Pods et al., 2013
Initial extracellular Na^+^ concentration	${\left[\text{Na}\right]}_{e}^{0}$	100	mM	Pods et al., 2013
Initial intracellular K^+^ concentration	${\left[\text{K}\right]}_{i}^{0}$	125	mM	Pods et al., 2013
Initial extracellular K^+^ concentration	${\left[\text{K}\right]}_{e}^{0}$	4	mM	Pods et al., 2013
Initial intracellular Cl^-^ concentration	${\left[\text{Cl}\right]}_{i}^{0}$	137	mM	Pods et al., 2013
Initial extracellular Cl^-^ concentration	${\left[\text{Cl}\right]}_{e}^{0}$	104	mM	Pods et al., 2013
Initial membrane potential	$\phi_{M}^{0}$	−67.74·10^−3^	V	
Initial HH gating value (Na^+^ activation)	m^0^	0.0379		Hodgkin and Huxley, 1952
Initial HH gating value (Na^+^ inactivation	h^0^	0.688		Hodgkin and Huxley, 1952
Initial HH gating value (K^+^ activation)	n^0^	0.276		Hodgkin and Huxley, 1952
Global time step	Δ*t*	1.0·10^−5^	s	
Local time step	Δt^*^	Δ*t*/25	s	

*The values are collected from Hodgkin and Huxley (1952), Hille (2001), and Pods et al. (2013). All units are reported in SI base units*.

#### 2.3.1. Model A: One Axon With a Passive Membrane Model

For Model A, we consider a two-dimensional domain $\Omega =\Omega_{i}\cup \Omega_{e}=\left[0,6.0\cdot 10^{-5}\right]\times \left[0,6.0\cdot 10^{-5}\right]$ m, with one intracellular domain (cell) $\Omega_{i}=\left[6.0\cdot 10^{-6},5.6\cdot 10^{-5}\right]\times \left[2.8\cdot 10^{-5},3.4\cdot 10^{-5}\right]$ m. We mesh this domain by dividing the domain into *n* × *m* rectangles, with Δ*x* = 6.0·10^−5^/*n* and Δ*y* = 3.0·10^−5^/*m*, and dividing each rectangle into two triangles by a diagonal, for a series of Δ*x* = Δ*y* = 2.0·10^−6^, 1.0·10^−6^, 5.0·10^−7^, 2.5·10^−7^ m. We model *I*_ch_ using the passive model, as described in section 2.1.4, and prescribe a synaptic input *I*_syn_ of the form

(37) $$\begin{array}{l} I_{\text{syn}}=g_{\text{syn}}H\left(x\right)e^{\frac{t-t_{0}}{\alpha }}\left(\phi_{M}-E_{\text{Na}}\right), \end{array}$$

where α is the synaptic time constant, *H*(*x*) = {1 for *x* ∈ *Z* and 0 elsewhere} for an interval *Z*. We let *Z* = [5.0 · 1.0^−5^, 1.0 · 10^−5^] m, and set *t*_0_ = 0, $g_{\text{syn}}=1.25\cdot 10^{3}$ S/m^2^. At the exterior boundary ∂Ω, we apply the boundary condition

(38) $$\begin{array}{l} {\text{J}}_{e}^{k}\cdot {\text{n}}_{e}=0,\text{at }\partial \Omega , \end{array}$$

describing that no ions can leave or enter the system.

#### 2.3.2. Model B: One Axon With a Passive Membrane Model and Non-physical Parameters

To evaluate the numerical accuracy of the mortar finite element scheme presented in section 2.2, we construct an analytical solution using the method of manufactured solutions (Roache, 1998). In particular, we let the analytical solution to (1)–(15) be given by:

(39) $$\begin{array}{l} \begin{array}{cc} {\text{[Na]}}_{i}^{e}=0.7+0.3\sin\left(2\pi x\right)\sin\left(2\pi y\right)\exp\left(-t\right), & \text{ in}\Omega_{i},\\ {\text{[Na]}}_{e}^{e}=1.0+0.6\sin\left(2\pi x\right)\sin\left(2\pi y\right)\exp\left(-t\right), & \text{ in}\Omega_{e},\\ {\text{[K]}}_{i}^{e}=0.3+0.3\sin\left(2\pi x\right)\sin\left(2\pi y\right)\exp\left(-t\right), & \text{ in}\Omega_{i},\\ {\text{[K]}}_{e}^{e}=1.0+0.2\sin\left(2\pi x\right)\sin\left(2\pi y\right)\exp\left(-t\right), & \text{ in}\Omega_{e},\\ {\text{[Cl]}}_{i}^{e}=1.0+0.6\sin\left(2\pi x\right)\sin\left(2\pi y\right)\exp\left(-t\right), & \text{ in}\Omega_{i},\\ {\text{[Cl]}}_{e}^{e}=2.0+0.8\sin\left(2\pi x\right)\sin\left(2\pi y\right)\exp\left(-t\right), & \text{ in}\Omega_{e},\\ \phi_{i}^{e}=\cos\left(2\pi x\right)\cos\left(2\pi y\right)\left(1+\exp\left(-t\right)\right), & \text{ in}\Omega_{i},\\ \phi_{e}^{e}=\cos\left(2\pi x\right)\cos\left(2\pi y\right), & \text{ in}\Omega_{e}, \end{array} \end{array}$$

with the passive model *I*_ch_ = ϕ_*M*_ and with *I*syn = 0. We assume that the parameter values all equal one: $C_{m}=D_{i}^{k}=D_{e}^{k}=F=G=R=1$, and that *K* = {Na^+^, K^+^, Cl^−^}. We consider a two-dimensional domain Ω = Ω_*i*_∪Ω_*e*_ = [0, 1] × [0, 1], with one intracellular domain Ω_*i*_ = [0.25, 0.75] × [0.25, 0.75]. The domain is meshed as for Model A (cf. section 2.3.1) for a series of *n* = *m* = 8, 16, 32, 64, 128, 256. In the numerical experiments for this test case, we initially let $\Delta t=\frac{1}{64}\cdot 10^{-5}$, and then quarter the timestep in each series. The errors are evaluated at $t=\frac{2}{64}\cdot 10^{-5}$.

#### 2.3.3. Model C: Multiple Axons With a Passive Membrane Model

For Model C, we define three different two-dimensional domains: (C1) a domain with one intracellular region (cell), (C2) a domain with two intracellular regions with a distance of 4.0·10^−6^m in the *y*-direction between the cells, and (C3) a domain with two intracellular regions with a distance of 1.0·10^−5^m in the *y*-direction between the cells. More precisely, we let

**Model C1:** $\Omega =\Omega_{i}\cup \Omega_{e}=\left[0,1.2\cdot 10^{-4}\right]\times \left[0,1.2\cdot 10^{-4}\right]$ m, $\Omega_{i}=\left[3.5\cdot 10^{-5},8.5\cdot 10^{-5}\right]\times \left[5.7\cdot 10^{-5},6.3\cdot 10^{-5}\right]$ m.
**Model C2:** $\Omega =\Omega_{i}^{1}\cup \Omega_{i}^{2}\cup \Omega_{e}=\left[0,1.2\cdot 10^{-4}\right]\times \left[0,1.2\cdot 10^{-4}\right]$ m, with two cells $\Omega_{i}^{1}=\left[3.5\cdot 10^{-5},8.5\cdot 10^{-5}\right]\times \left[5.2\cdot 10^{-5},5.8\cdot 10^{-5}\right]$ m and $\Omega_{i}^{2}=\left[3.5\cdot 10^{-5},8.5\cdot 10^{-5}\right]\times \left[6.2\cdot 10^{-5},6.8\cdot 10^{-5}\right]$ m.
**Model C3:** Ω as in Model C2 but with $\Omega_{i}^{1}=\left[3.5\cdot 10^{-5},8.5\cdot 10^{-5}\right]\times \left[4.9\cdot 10^{-5},5.5\cdot 10^{-5}\right]$ m and $\Omega_{i}^{2}=\left[3.5\cdot 10^{-5},8.5\cdot 10^{-5}\right]\times \left[6.5\cdot 10^{-5},7.1\cdot 10^{-5}\right]$ m.

The ion channel currents ${\text{I}}_{\text{ch}}^{k}$ are modeled using the passive model described in section 2.1.4. The synaptic input current model (37) is applied with $g_{\text{syn}}=1.25\cdot 10^{3}$ S/m^2^, *t*_0_ = 0, and with *Z* = [3.5·10^−5^, 4.0·10^−5^] m for Model C1, $Z_{1}=\left[6.0\cdot 10^{-5},6.5\cdot 10^{-5}\right]$ m for Model C2, and $Z_{2}=\left[5.5\cdot 10^{-5},6.0\cdot 10^{-5}\right]$ m for Model C3. At the exterior boundary ∂Ω, we apply the boundary condition (38). In order to compare the KNP-EMI and the EMI framework, we set the bulk conductivity σ_*r*_ in the EMI model by (5) with initial values ${\left[k\right]}_{r}^{0}$ for the ion concentration [*k*]_*r*_. Note that σ_*r*_ will generally change over time.

#### 2.3.4. Model D: Axon Bundle With Active Hodgkin-Huxley Membrane Model

For Model D, we consider a domain $\Omega =\Omega_{i}^{1}\cup \cdots \cup \Omega_{i}^{9}\cup \Omega_{e}=\left[0,4.0\cdot 10^{-4}\right]\cdot \left[0,1.4\cdot 10^{-6}\right]\times \left[0,1.4\cdot 10^{-6}\right]$ m, where 9 cuboidal cells of size 3.9·10^−4^ × 2.0·10^−7^ × 2.0·10^−7^ m are placed uniformly throughout Ω (cf. Figure 1). The distance between the cells is 1.0·10^−7^ m. The domain is meshed as in section 2.3.1 with Δ*y* = Δ*z* = 1.0·10^−7^ m and Δ*x* = 1.25·10^−6^ m. The ion channel currents are modeled using the Hodgkin-Huxley model as described in section 2.1.4. An action potential is induced every 20 ms throughout the simulations by applying the synaptic input current model (37) with *g*_syn_ = 40 S/m^2^, α = 0.002 s, and *t*_0_ = 0, 0.02, 0.04 s. We ran two sets of simulations: (1) stimulating, i.e., applying the synaptic current to the membrane of, the middle axon only (axon A in Figure 1), and (2) stimulating the 8 axons around axon A (axons B,C in Figure 1). At the exterior boundary, we apply the boundary condition (38).

## 3. Results

We here present results from numerical experiments using the KNP-EMI framework and the numerical method presented above. We start by assessing the accuracy (Model A and B) and performance (Model A and D) of the numerical method. Next, we compare the KNP-EMI and EMI frameworks in idealized 2D axons (Model C), before we finally investigate ephaptic coupling in unmyelinated axons bundles (Model D). Note that the values in this section are given in physiologically reasonable units for the sake of readability; i.e., the results have been converted from the SI base units, e.g., seconds to milliseconds.

### 3.1. Numerical Verification and Accuracy

To evaluate the numerical accuracy and convergence of the proposed numerical approach, we consider two sets of experiments. First, we examine the convergence of the model under mesh refinement by visual inspection. Second, we perform a formal convergence analysis for a smooth test case with manufactured solution.

#### 3.1.1. Inspection of Convergence Under Mesh Refinement

**Figure 3:**
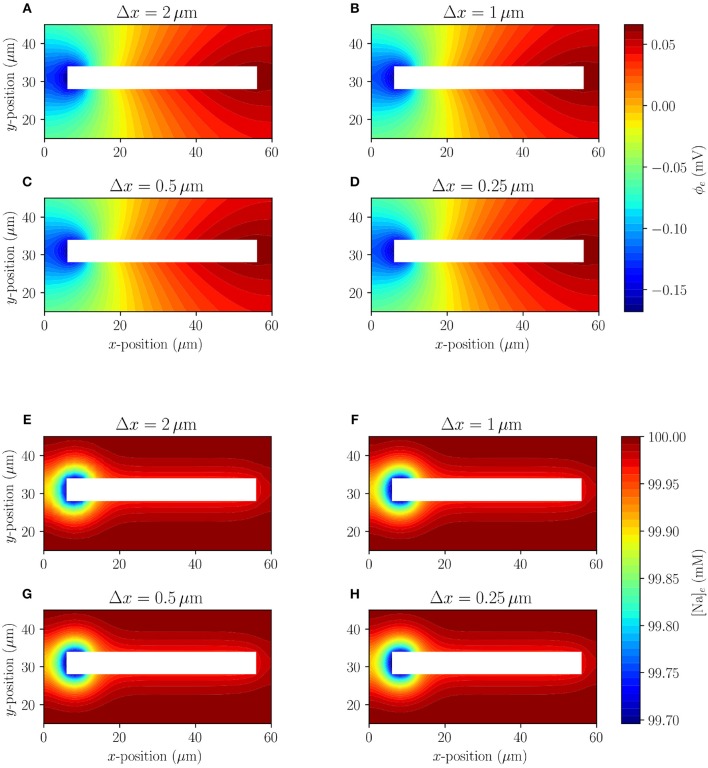
Model A: Comparison (under mesh refinement) of extracellular potential **(A–D)** and extracellular sodium concentration **(E–H)** in the surroundings of a single simplified axon at *t* = 10 ms.

The extracellular potential and sodium (Na^+^) concentration of Model A for four different mesh resolutions are shown at *t* = 10 ms in Figure 3. The system quickly (after 3 ms) reaches a semi-steady state where the membrane potential does not change notably over time, but there is a slow exchange of sodium (Na^+^) and potassium (K^+^) ions due to the leak currents. The extracellular sodium concentration does not appear to change visibly under mesh refinement, and the extracellular potential seems to reach a converged state for the finest mesh resolution. More precisely, the mean relative difference (over time) between the solutions for the finest mesh (Δ*x* = 0.25) and the coarser mesh resolutions Δ*x* = 2.0, Δ*x* = 1.0, and Δ*x* = 0.5 are 4.8·10^−5^, 4.6·10^−6^, and 1.6·10^−6^ for [Na]_*e*_, respectively, and 2.0·10^−2^, 2.3·10^−3^, and 4.3·10^−4^ for ϕ_*e*_, respectively, at the point (4, 31). We conclude the differences between the solutions are small and decreasing, indicating convergence of the method.

#### 3.1.2. Convergence Rates of Numerical Solutions

Using Model B, we analyzed the rates of convergence for the approximations of all solution variables under refinement in space and time. Based on properties of the approximation spaces and the time discretization, the optimal theoretical rate of convergence is 1 in the *H*^1^-norm and 2 in the *L*^2^-norm and the broken *L*^2^-norm. Our numerical findings (Table 2) are in agreement with the theoretically optimal rates. We observe second order convergence in the *L*^2^-norm for the approximation of the extracellular and intracellular concentrations and potentials, and first order convergence in the *H*^1^-norm. For the transmembrane ionic current *I*_*M*_, we observe a convergence rate of 1.5 in the broken *L*^2^-norm. The loss of convergence of ~0.5 for *I*_*M*_ is likely due to a lack of smoothness of the interface in the test domain.

**Table 2:** Approximation errors (with convergence rates in parenthesis) for the extracellular and intracellular concentrations and potentials, and transmembrane current, under simultaneous refinement in time and space.

***n***	**_**[Na]_*i*_−[Na]_*i, h*_0**_**	**_**[Na]_*e*_−[Na]_*e, h*_0**_**	**_**[Na]_*i*_−[Na]_*i, h*_1**_**	**_**[Na]_*e*_−[Na]_*e, h*_1**_**
8	9.01E-03 (—)	3.12E-02 (—)	2.54E-01 (—)	8.80E-01 (—)
16	2.33E-03 (1.95)	8.08E-03 (1.95)	1.30E-01 (0.97)	4.50E-01 (0.97)
32	5.88E-04 (1.99)	2.04E-03 (1.99)	6.53E-02 (0.99)	2.26E-01 (0.99)
64	1.47E-04 (2.00)	5.10E-04 (2.00)	3.27E-02 (1.00)	1.13E-01 (1.00)
128	3.69E-05 (2.00)	1.28E-04 (2.00)	1.64E-02 (1.00)	5.67E-02 (1.00)
256	9.22E-06 (2.00)	3.21E-05 (1.99)	8.18E-03 (1.00)	2.86E-02 (0.99)
***n***	_**[K]_*i*_−[K]_*i, h*_0**_	_**[K]_*e*_−[K]_*e, h*_0**_	_**[K]_*i*_−[K]_*i, h*_1**_	_**[K]_*e*_−[K]_*e, h*_1**_
8	9.01E-03 (—)	1.04E-02 (—)	2.54E-01 (—)	2.93E-01 (—)
16	2.33E-03 (1.95)	2.69E-03 (1.95)	1.30E-01 (0.97)	1.50E-01 (0.97)
32	5.88E-04 (1.99)	6.79E-04 (1.99)	6.53E-02 (0.99)	7.54E-02 (0.99)
64	1.47E-04 (2.00)	1.70E-04 (2.00)	3.27E-02 (1.00)	3.78E-02 (1.00)
128	3.69E-05 (2.00)	4.25E-05 (2.00)	1.64E-02 (1.00)	1.89E-02 (1.00)
256	9.22E-06 (2.00)	1.20E-05 (1.82)	8.18E-03 (1.00)	1.02E-02 (0.89)
***n***	_**[Cl]_*i*_−[Cl]_*i, h*_0**_	_**[Cl]_*e*_−[Cl]_*e, h*_0**_	_**[Cl]_*i*_−[Cl]_*i, h*_1**_	_**[Cl]_*e*_−[Cl]_*e, h*_1**_
8	1.80E-02 (—)	4.16E-02 (—)	5.08E-01 (—)	1.17E+00 (—)
16	4.67E-03 (1.95)	1.08E-02 (1.95)	2.60E-01 (0.97)	6.00E-01 (0.97)
32	1.18E-03 (1.99)	2.72E-03 (1.99)	1.31E-01 (0.99)	3.02E-01 (0.99)
64	2.95E-04 (2.00)	6.82E-04 (2.00)	6.54E-02 (1.00)	1.51E-01 (1.00)
128	7.38E-05 (2.00)	1.71E-04 (1.99)	3.27E-02 (1.00)	7.56E-02 (1.00)
256	1.84E-05 (2.00)	4.48E-05 (1.93)	1.64E-02 (1.00)	3.85E-02 (0.97)
***n***	_****ϕ**_*i*_−**ϕ**_*i, h*_0**_	_****ϕ**_*e*_−**ϕ**_*e, h*_0**_	_****ϕ**_*i*_−**ϕ**_*i, h*_1**_	_****ϕ**_*e*_−**ϕ**_*e, h*_1**_
8	9.37E-02 (—)	6.60E-02 (—)	1.69E+00 (—)	1.42E+00 (—)
16	2.52E-02 (1.90)	1.80E-02 (1.87)	8.66E-01 (0.96)	7.42E-01 (0.94)
32	6.41E-03 (1.97)	4.63E-03 (1.96)	4.35E-01 (0.99)	3.76E-01 (0.98)
64	1.61E-03 (2.00)	1.17E-03 (1.99)	2.18E-01 (1.00)	1.89E-01 (1.00)
128	3.96E-04 (2.02)	2.95E-04 (1.98)	1.09E-01 (1.00)	9.44E-02 (1.00)
256	9.04E-05 (2.13)	8.14E-05 (1.86)	5.45E-02 (1.00)	4.74E-02 (0.99)
***n***	_***I*_*M*_−*I*_*M, h*_0, **Γ****_			
8	7.03E+00 (—)			
16	2.54E+00 (1.47)			
32	8.93E-01 (1.51)			
64	3.14E-01 (1.51)			
128	1.11E-01 (1.50)			
256	3.94E-02 (1.49)			

*Approximation errors are measured at $t=\frac{2}{64}\cdot 10^{-5}$, i.e., e.g., ||[Na]_r_−[Na]_r, h_||_0_ = ||[Na]_r_(t)−[Na]_r, h_(t)||_0_ and similarly for all reported values. The largest time step is Δt = 1/64·10^−5^; the time step was quartered in each refinement level*.

#### 3.1.3. Effect of Boundary Conditions

**Figure 4:**
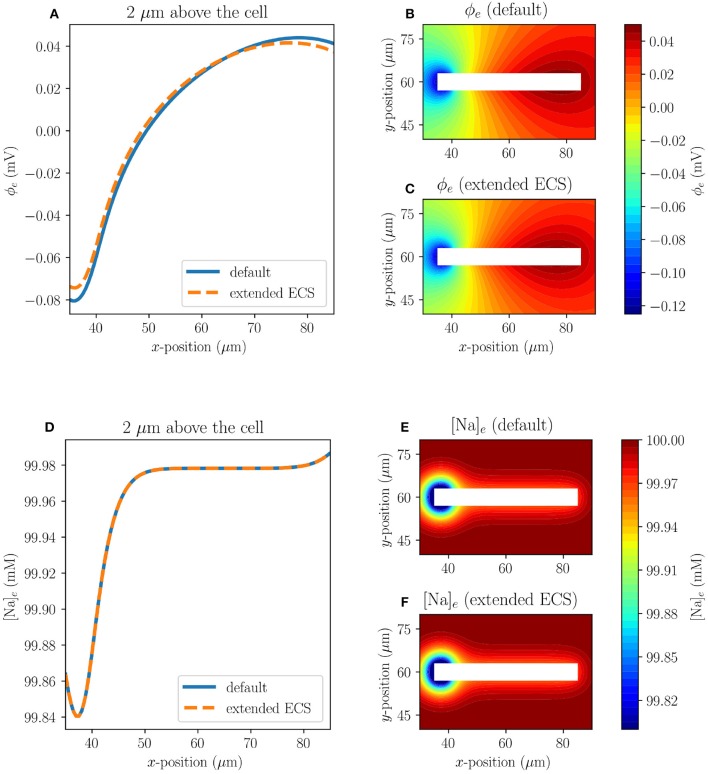
Comparison of results in Model C1 with the default size of ECS (120 × 120 μm), and with an extended ECS (240 × 240 μm) at *t* = 10 ms. The upper panels display the ECS potential, both at 2μm above the axon **(A)** and in the surrounding ECS for the default model **(B)** and for the model with extended ECS **(C)**. The lower panels display the ECS sodium concentration, both at 2μm above the axon **(D)** and in the surrounding ECS for the default model **(E)** and for the model with extended ECS **(F)**.

To examine whether the size of the extracellular space (ECS) affects the solution near the axon, we consider Model C1 with both the default size of the ECS (120 × 120 μm) and with an extended ECS (240 × 240 μm). The axon is placed in the same position in both cases. The two cases do not differ substantially in the extracellular Na^+^ concentrations or the extracellular potentials near the axon (Figure 4). The maximal difference between the default model and the model with extended extracellular space, measured 2μm above the axon at *t* = 10 ms, for ϕ_*e*_ and [Na]_*e*_ are 6.33·10^−3^ mV and 5.72·10^−6^ mM, respectively.

### 3.2. Numerical Performance

To evaluate the performance and scalability of the implementation of the presented framework, we consider an additional set of experiments measuring the memory usage and CPU timings for simulations of Model A (2D) and D (3D). We observe that the memory usage increases sublinearly with the system size: increasing the system size by a factor of four for Model A leads to an increase in memory of a factor 1−3 (Table 3). We observe that the CPU time for the simulations grows superlinearly with the system size: increasing the system size by a factor of four for Model A leads to an increase in total simulation time of a factor 3−5 (Table 3). This behavior is expected as the linear systems are solved using a direct solver. The total simulation time is dominated by the cost of finite element assembly and linear solves (70–94%). For small system sizes, the time required for finite element assembly is comparable to that of the linear solves. However, for larger system sizes, and especially in 3D, the linear solution time dominates the total simulation cost.

**Table 3:** CPU timings and memory usage for different KNP-EMI scenarios (Models A and D).

**Model**	**Δ*x*(μm)**	**Size**	**Memory (MiB)**	**T_*A*_ (s)**	**T_*S*_ (s)**	**T (s)**	**# N**
A	2	4,125	34	22.9	24.8	67.2	1,000
A	1	15,445	43	26.6	134	191	1,000
A	0.5	59,685	113	37.8	662	777	1,000
A	0.25	234,565	241	98.5	4,168	4,604	1,000
D		401,671	300	86.6	7,190	7,740	100

Δ*x: mesh resolution (for Model A), Size, linear system size (number of degrees of freedom); Memory, Maximal memory usage of simulation relative to baseline. T_A_, CPU time for finite element assembly; T_S_, CPU time for linear system solution; T, Total CPU time for simulation; N, number of timesteps*.

### 3.3. Comparison of the KNP-EMI and EMI Frameworks in Idealized Axons

The KNP-EMI framework extends the EMI framework by explicitly modeling the ionic concentrations and incorporating ionic electrodiffusion. A key question is when and to what extent the solutions from the two (KNP-EMI and EMI) frameworks differ. To compare the two frameworks, we consider three models (Model C1, C2, and C3) and compare the corresponding solution of the KNP-EMI equations (the KNP-EMI solution) with the solution of the EMI equations (the EMI solution).

**Figure 5:**
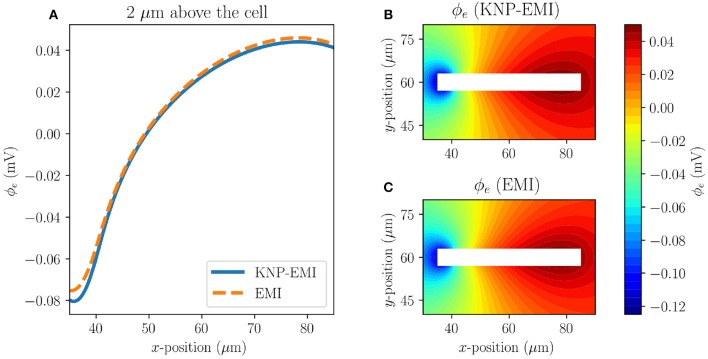
A comparison of the KNP-EMI and the EMI frameworks using Model C1 at *t* = 10 ms. Extracellular potentials measured 2μm above the cell **(A)**. Extracellular potentials from the KNP-EMI **(B)** and the EMI framework **(C)** surrounding the cell.

We first consider the extracellular potential resulting from stimulating a single axon (Model C1) using the KNP-EMI and EMI frameworks (Figure 5). We observe that the KNP-EMI and EMI solutions are qualitatively very similar: an extracellular potential difference of ~0.12 mV along the length of the axon develops in both (Figures 5A–C).

**Figure 6:**
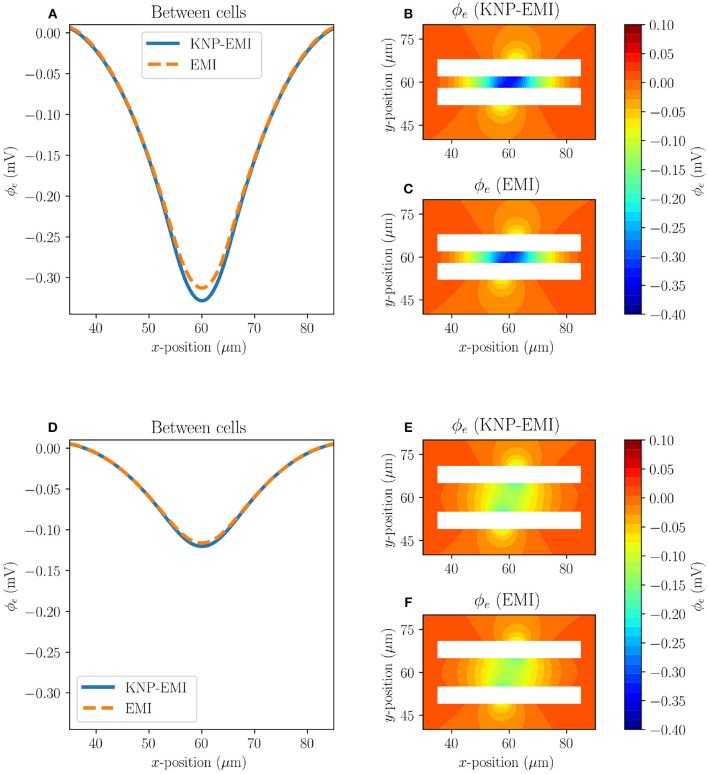
A comparison of the KNP-EMI and the EMI frameworks using Model C2 and C3 at *t* = 10 ms. The extracellular potentials from Model C2 **(A)** and C3 **(D)** on the midline between the neurons (*y* = 60μm). The extracellular potentials surrounding the cells as calculated by KNP-EMI **(B)** and EMI **(C)** using Model C2, and by KNP-EMI **(E)** and EMI **(F)** using Model C3.

Next, we compare the extracellular potentials resulting from stimulating two neighboring axons (Model C2 and C3) using the KNP-EMI and the EMI frameworks (Figure 6). The two models differ by the distance between the axons. For Model C2, we again observe that the KNP-EMI and EMI solutions match closely, but differ locally. The maximal difference between the extracellular potential solutions is 0.016 mV (Figures 6A–C). For Model C3, we observe the analogous behavior, but note that the extracellular field is weaker than for Model C2 (Figures 6D–F).

### 3.4. Ephaptic Coupling in Unmyelinated Axon Bundles

We now turn to explore the effect of ephaptic coupling in an idealized axon bundle with 9 axons using the KNP-EMI framework. We consider two sets of simulations using Model D: (1) stimulating, i.e., applying the synaptic current to the membrane of, the middle axon only (axon A, Figure 1), and (2) stimulating the 8 axons around axon A (axons B,C, Figure 1).

#### 3.4.1. Electrodiffusion Effects in Unmyelinated Axon Bundles

**Figure 7:**
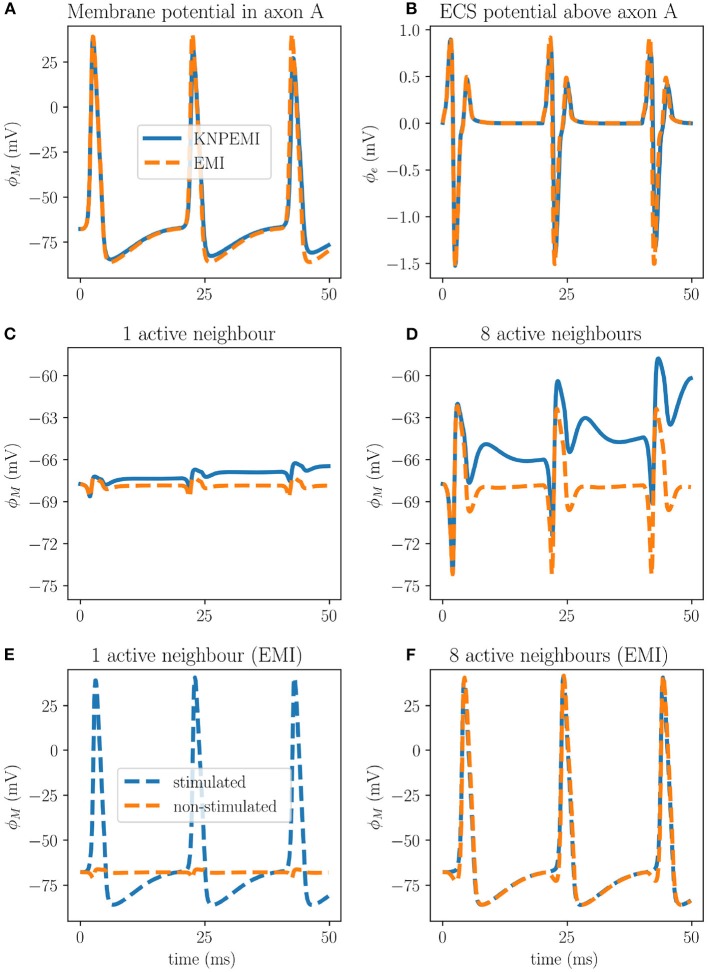
Effects of ephaptic coupling in a bundle of axons at *x* = 200 μm. The membrane potential (ϕ_*M*_) of axon A **(A)**, and the extracellular potential (ϕ_*e*_) measured 0.05 μm away from the membrane of axon A **(B)** during stimuli of axon A only. Ephaptic coupling measured in axon B when only axon A is stimulated **(C)**, and measured in axon A when all peripheral axons (B–C) are stimulated **(D)**. Setting σ_*i*_ = 1.0 S/m and σ_*e*_ = 0.1 S/m in the EMI framework increases the ephaptic coupling to the point where simultaneous action potentials in all eight surrounding axons will induce an action potential in the central axon **(F)**. However, only stimulating the middle axon (A) will not induce action potentials in the peripheral axons **(E)**.

To investigate ephaptic coupling, we first apply a synaptic current to stimulate the cell membrane of the middle axon of the axon bundle (axon A, Figure 1, Model D). The synaptic current induces a series of action potentials in axon A and also induces substantial changes in the surrounding extracellular potential (Figures 7A,B). The extracellular potential fluctuations further spread to axon B. However, the ephaptic effect on the membrane potential in axon B is relatively small (1–2 mV), and is not sufficient to reach the threshold for inducing an action potential (Figure 7C).

The ephaptic effect is stronger if we simultaneously stimulate the cell membranes of all eight peripheral axons (axons B–C). Again, we observe a series of action potentials in the eight stimulated axons. Moreover, the combined ephaptic currents have a pronounced excitatory effect on axon A, but again fail to induce an action potential there (Figure 7D).

**Figure 8:**
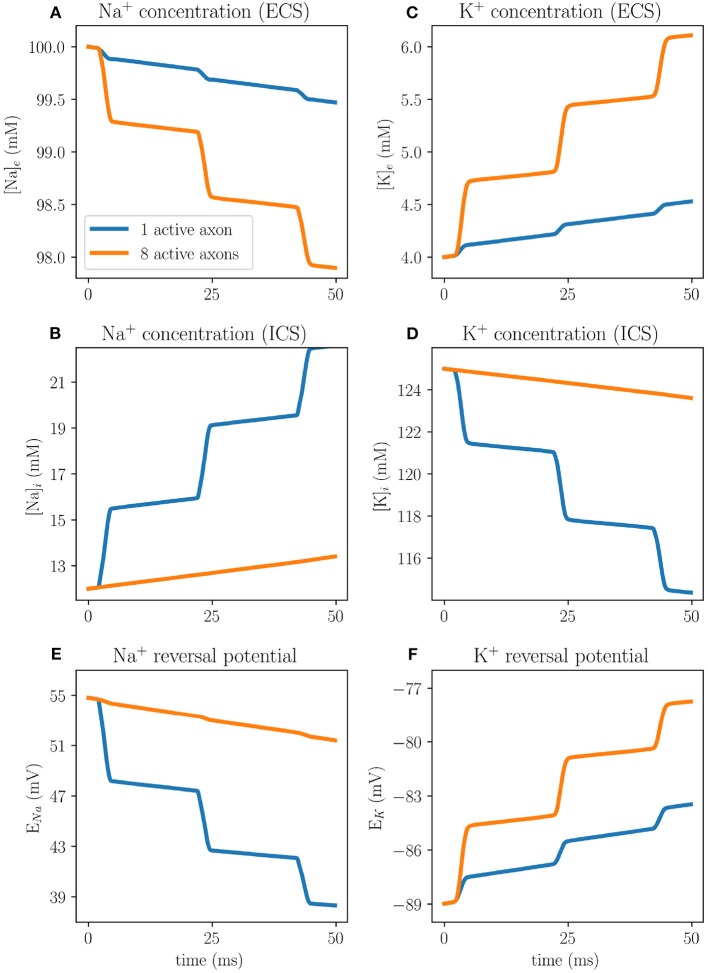
Ion concentration dynamics in an axon bundle measured at the middle axon (A) at *x* = 200 μm using the KNP-EMI framework, both when middle axon (A) is stimulated, and when all peripheral axons (B–C) are stimulated. Extracellular sodium **(A)** and extracellular potassium **(B)** concentrations evaluated 0.05 μm away from axon A. Intracellular sodium **(C)** and intracellular potassium **(D)** concentrations evaluated at the center of axon A. Reversal potentials for sodium **(E)** and potassium **(F)** at the membrane of axon A.

The difference between the EMI and KNP-EMI simulations are due to the time evolution of the intracellular and extracellular ion concentrations, accounted for by the KNP-EMI model but not by the EMI model. For each action potential fired, the Nernst potential will change due to alterations in the ionic concentrations using the KNP-EMI framework (Figure 8), whereas in the EMI framework the Nernst potential is constant.

Our predictions differ from those made in a similar study by Bokil et al. (2001), who found that a single active neighbor can induce action potentials in all nearby axons. We hypothesize that the main explanation for these differences is that the bulk conductivities differ between the two studies. Here, in the KNP-EMI framework, the bulk conductivities are functions of the ion concentrations [cf. (5)]. Using realistic values for the intra- and extracellular ion concentrations, we obtained bulk conductivities values of σ_*i*_≈2.01μS/μm and σ_*e*_≈1.31 S/m. In contrast, Bokil et al. set the bulk conductivities as free parameters, with σ_*i*_ = 1S/m and σ_*e*_ = 0.1S/m as the corresponding effective bulk conductivities in the EMI model. Tveito et al. (2017b) found that the ephaptic current was inversely proportional to σ_*e*_, which suggests that the ephaptic current was more than seven times stronger in Bokil et al. (2001) than here.

In light of this, we repeated the simulations of the EMI model using the lower effective bulk conductivity values (σ_*i*_ = 1 S/m and σ_*e*_ = 0.1S/m). In this case, simultaneous stimulation of the 8 peripheral axons (B–C) induced an action potential in axon A (Figure 7F). Stimulation of axon A alone did not induce an action potential in the 8 peripheral axons (Figure 7E).

## 4. Discussion

We have presented a finite element-based numerical method for a revised mathematical model of ionic electrodiffusion with explicit geometrical representation of the extracellular space, the intracellular space and the cell membrane. Our numerical scheme is based on the mortar finite element method and is capable of efficiently handling complex geometries in one, two, or three spatial dimensions. Our numerical investigations demonstrate that the scheme is accurate and yields optimal convergence rates in the relevant norms.

Further, we compared the KNP-EMI framework and the EMI framework by computationally studying (i) extracellular fields surrounding passive idealized axons, and (ii) membrane potentials in a bundle of unmyelinated axons under Hodgkin-Huxley membrane mechanisms. The potentials predicted by the two frameworks are essentially identical during the first period (~5 ms) of the simulations, but the predictions later differ due to changes in ion concentrations (only accounted for by the KNP-EMI framework). We note that the strongest ephaptic coupling is due to changes in the Nernst potentials (ionic ephaptic coupling), and not via extracellular potentials (electric ephaptic coupling).

The predictions of ephaptic coupling made in this study differs from those made by Bokil et al. (2001) using cable theory. This discrepancy is likely due to differences in the extracellular bulk conductivities. Indeed, an important difference between geometrically explicit frameworks (e.g., PNP, EMI, and KNP-EMI) and homogenized frameworks (e.g., cable theory) is the interpretation of the bulk conductivities σ_*i*_ and σ_*e*_. In homogenized frameworks based on volume-conductor theory, the bulk conductivity σ is interpreted as the tissue average, i.e., the effective bulk conductivity for currents propagating over distances in brain tissue (Holt and Koch, 1999; Pettersen and Einevoll, 2008; Reimann et al., 2013). Importantly, this tissue-averaged bulk conductivity is smaller than the actual conductivity of the extracellular solution, largely due to the fact that the extracellular space only constitutes about 20% of the total tissue volume. On the other hand, in the KNP-EMI framework, the bulk conductivities are defined in terms of the local ion concentrations and will thus vary consistently across the domain.

The KNP-EMI framework is comparable to the PNP framework (Lopreore et al., 2008; Pods et al., 2013; Holcman and Yuste, 2015; Cartailler et al., 2017a,b; Sacco et al., 2017) as both frameworks can account for the explicit morphology of neural tissue (Noguchi et al., 2005; Biess et al., 2007). The difference between the frameworks is in the way that the electrical potential ϕ is computed. In the more physically detailed PNP framework, ϕ is computed from the Poisson equation ∇^2^ϕ = −ρ/ϵ, where ρ is the charge density and ϵ is the permittivity of the medium. In neural tissue, the charge relaxation time, i.e., the typical time-scale that ρ varies on, is in the order of 1 ns, and most of the local net charge density is resolved in nanometer thick layers surrounding neuronal membranes (Grodzinsky, 2011; Gratiy et al., 2017). Hence, simulations on the PNP framework requires a spatiotemporal resolution smaller than nanoseconds and nanometers, and become unstable otherwise. In contrast, the KNP-EMI framework circumvents the need for explicit modeling of charge accumulation near the membrane by assuming bulk electroneutrality, so that all net charge is associated as a membrane charge and not resolved spatially; that is, the membrane interface conditions (6)–(15) ensure that the mesh elements bordering the membrane contain the charges consistent with the membrane potential, regardless of mesh size. The electroneutrality condition has been shown to be a good approximation on spatiotemporal scales larger than micrometers and microseconds (Pods, 2017; Solbrå et al., 2018), and the application of it results in a more numerically stable framework for coarser time and space resolutions, allowing for longer simulations on larger domains. The differences between the PNP framework and (bulk-) electroneutral frameworks, such as KNP have been discussed extensively in previous works (Mori and Peskin, 2009; Pods, 2017; Solbrå et al., 2018).

An example of a phenomenon where large ion concentration changes in brain tissue build up over time, is (cortical) spreading depression. During spreading depression, the extracellular K^+^ concentration can change from a basal level of 3–5 mM to peak values at tens of mM over a period of several minutes (Somjen, 2001). As such, we advocate that the KNP-EMI model would be suitable for studying cellular level aspects of spreading depression computationally. However, for simulations of longer duration (>50 ms), the membrane mechanism model should be chosen carefully. The Hodgkin-Huxley formalism used in this paper to describe the membrane mechanisms does not account for the effect of ion pumps and co-transporters, which generally will strive to restore concentrations to baseline. As a consequence, the concentration changes taking place in our simulations are likely overestimates of what could be expected in a real biological system. Adding ion pumps and co-transporters to the membrane model would be relatively straightforward (Hübel and Dahlem, 2014; Hübel et al., 2014).

In conclusion, the KNP-EMI framework presented in this paper allows for detailed computational studies of the interplay between ion movement, membrane mechanisms and electrical potential in healthy neural tissue and under pathological conditions. The computational expense of KNP-EMI simulations compared to, e.g., homogenized models calls for further research into efficient and scalable solution methods.

## Data Availability Statement

The datasets analyzed for this study are publicly available and can be found at the following source: https://zenodo.org/record/3492075#.XahQOhh9g5k.

## Author Contributions

AE, AS, GE, GH, and MR designed the study, derived the mathematical model and numerical method, and wrote the manuscript. AE and AS developed the software implementation and performed the numerical experiments.

### Conflict of Interest

The authors declare that the research was conducted in the absence of any commercial or financial relationships that could be construed as a potential conflict of interest.
